# Supplementary figures and images for: Versatile and flexible microfluidic qPCR test for high-throughput SARS-CoV-2 and cellular response detection in nasopharyngeal swab samples
Source: PLoS One. 2021 Apr 14;16(4):e0243333. doi: 10.1371/journal.pone.0243333 (PMC8046349; doi:10.1371/journal.pone.0243333)

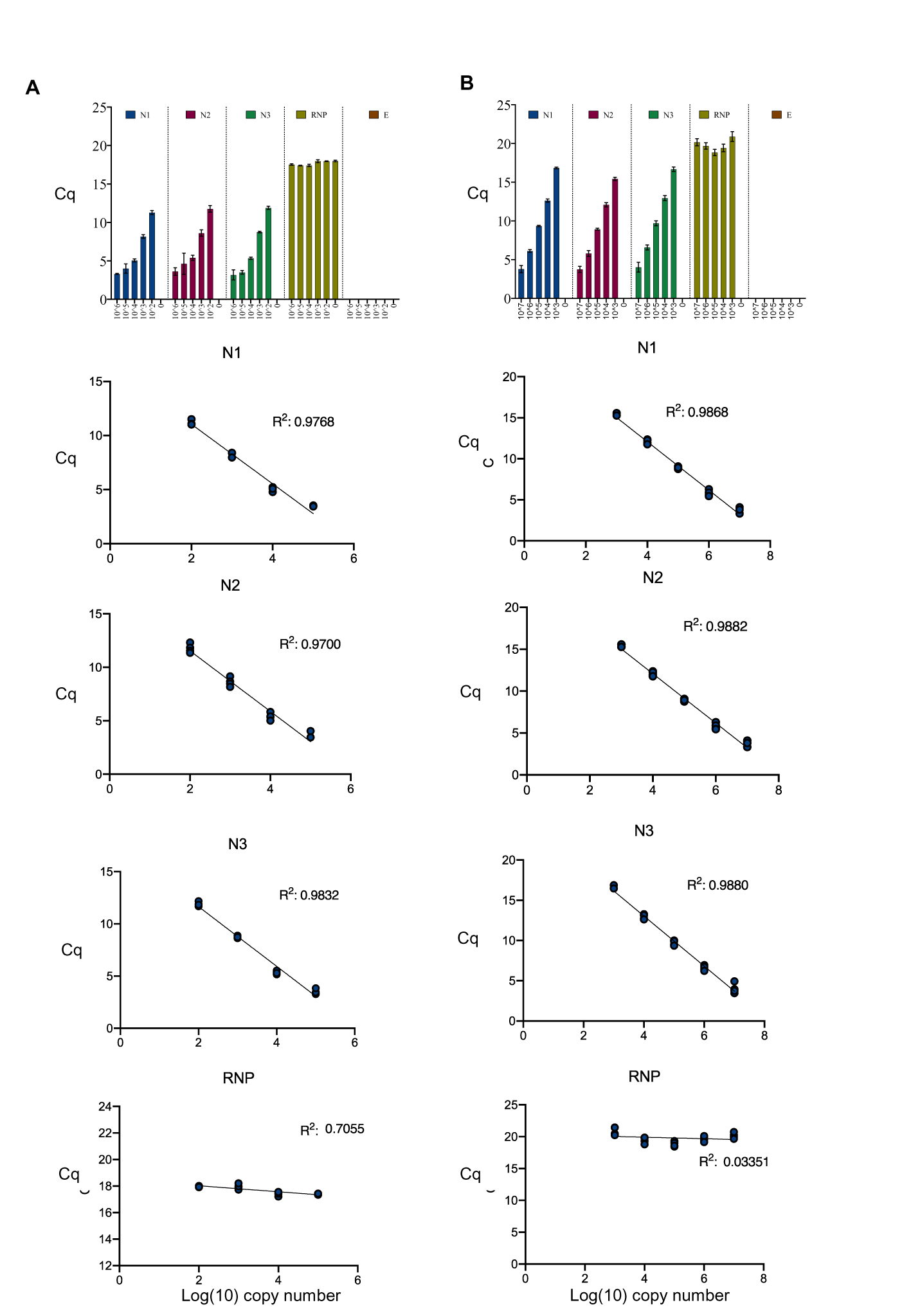

Supplement: S1 Fig — A ten-fold serial dilution ranging from 1 to 10–6 was prepared from the stock solution of the in vitro-transcribed N gene and supplemented with 2 ng/μL of total RNA from HEK 293 Cells. Reverse Transcription was performed followed by 15 cycles of pre-Amplification and 30 cycles of qPCR. The RT-qPCR reaction was performed without (A) or with (B) a RNA purification step. Linear regression was performed by logarithmic plots of transcript copy number against Cq value. We observed a good correlation according Cq linear regression curves according to dilution for the three viral CDC primers/probe sets (N1, N2, N3). No Cq value has been detected for the E primers/probe. RNP, used as internal control, shows constant detection of Cq value, suggesting a good performance of the qPCR. (TIF) [file pone.0243333.s001.tif]

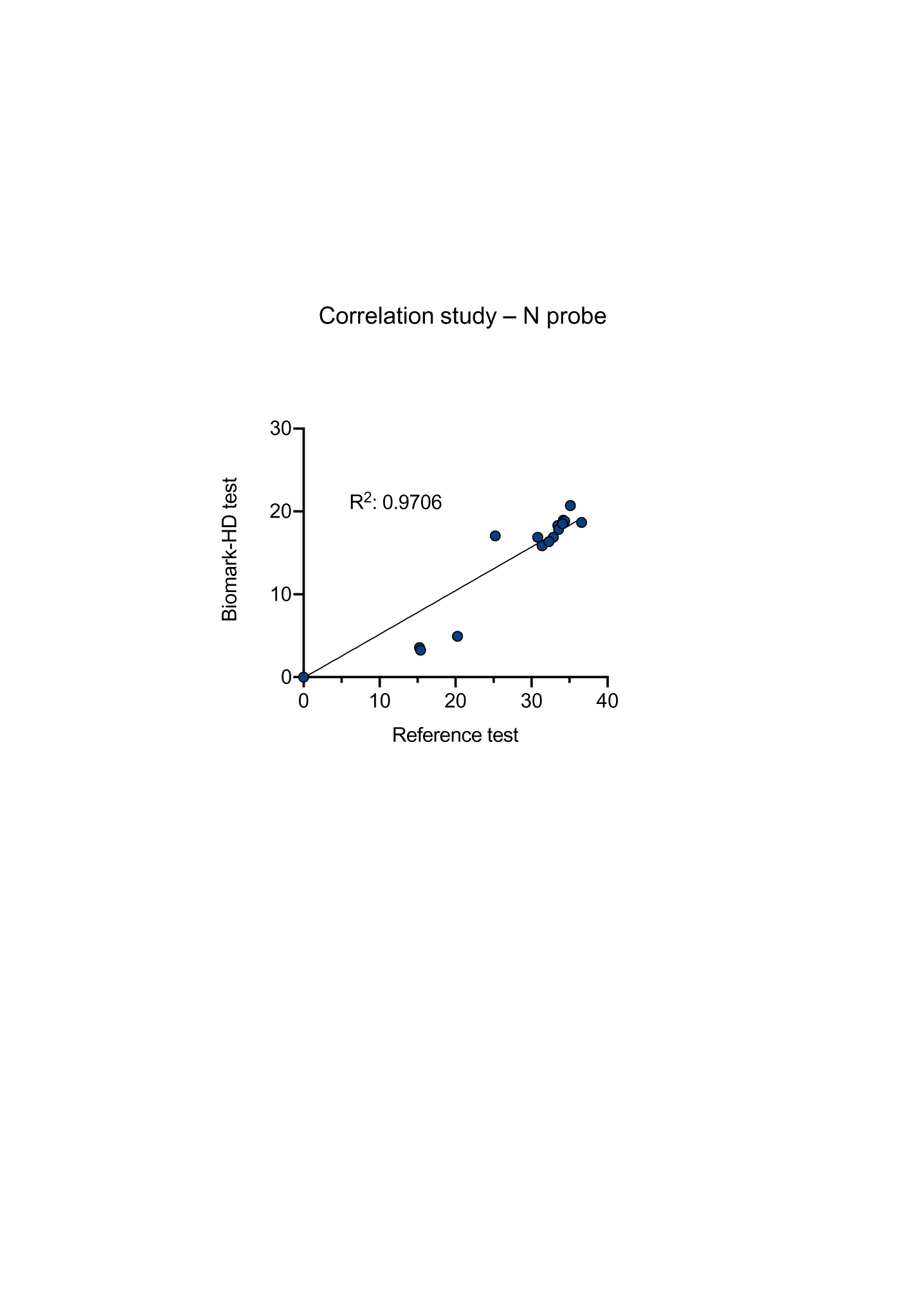

Supplement: S2 Fig — The correlation of the Cq values obtained for the N primers/probe (BiomarkTM -HD) and the GeneFirst COVID-19 detection kit is presented. (TIF) [file pone.0243333.s002.tif]

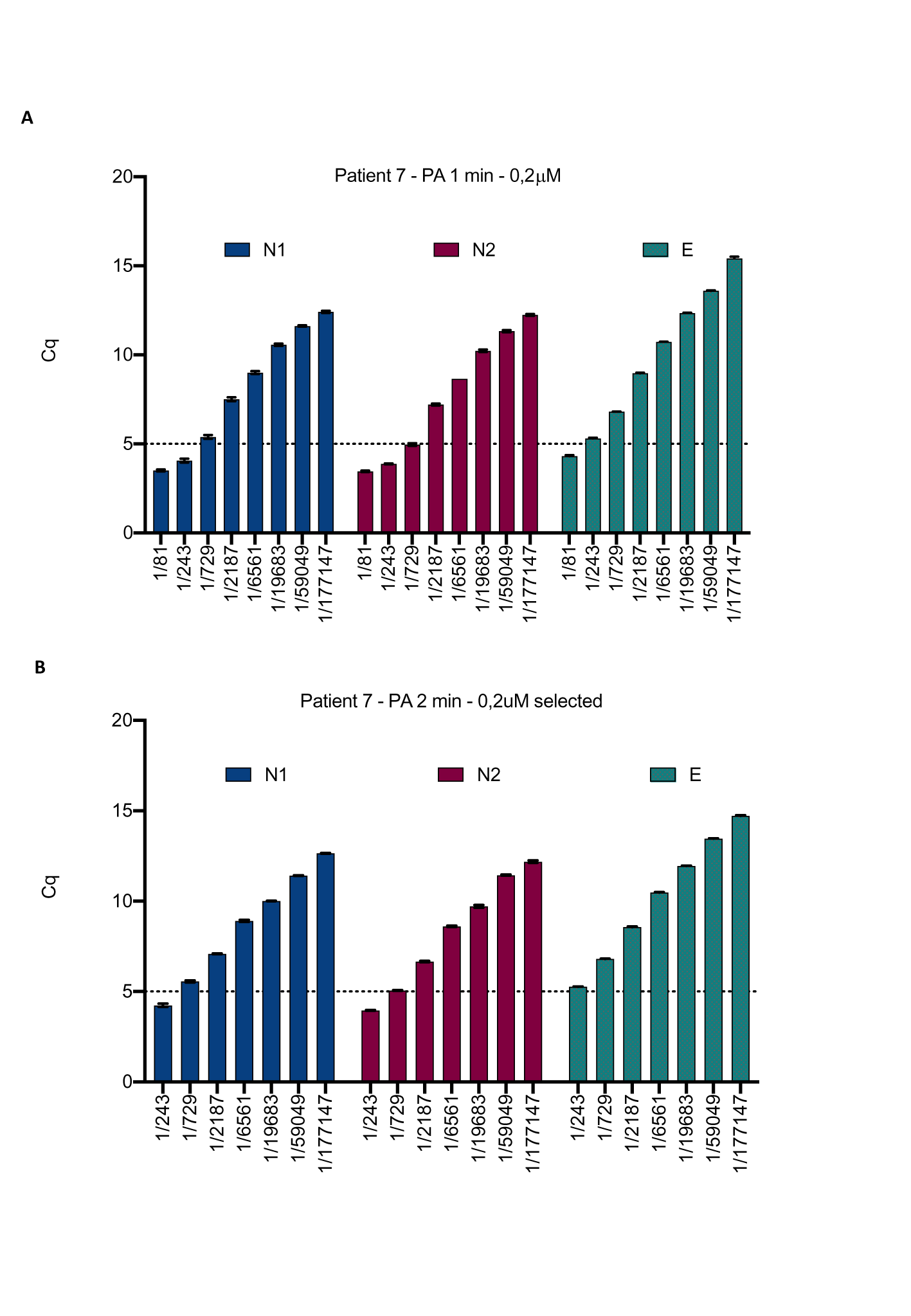

Supplement: S3 Fig — The elongation time used in the preamplification reaction was reduced to 1 min at 60°C (A) from 2 min at 60°C (B) using diluted total RNA from a SARS-nCov2 positive patient sample. (TIF) [file pone.0243333.s003.tif]

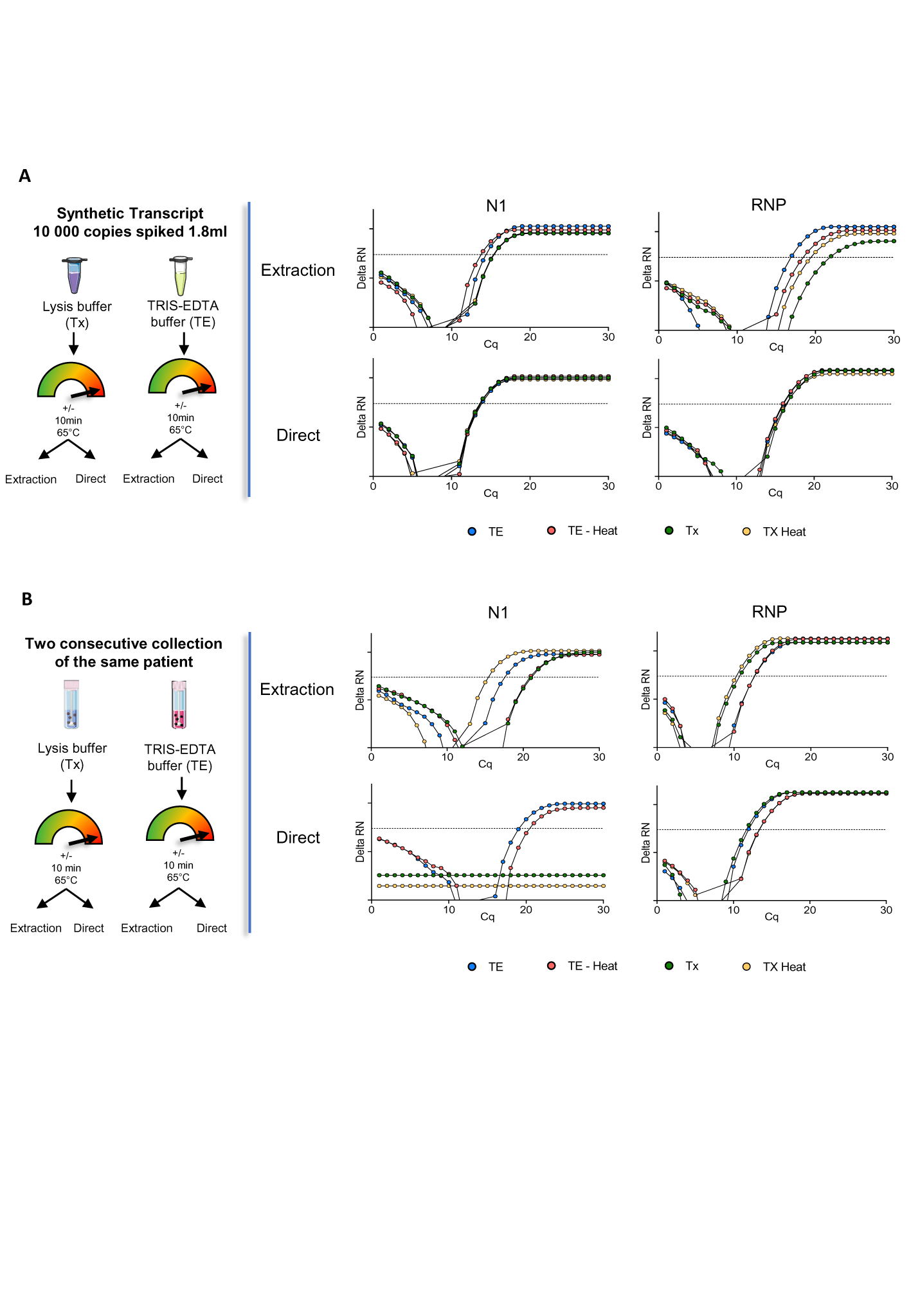

Supplement: S4 Fig — A. In vitro-transcribed viral N gene was added to either a Triton X-100 containing lysis buffer (Tx) or to TE buffer (TE). The samples were heated or not at 65°C for 10 min. RNA extraction was performed or not (direct) and N1 or RNP levels were determined by RT-qPCR using the BiomarkTM -HD system. B. A similar protocol as in A was used but the starting material were two consecutive sample collection from the same patient processed either in a Triton X-100 containing lysis buffer or to TE buffer. (TIF) [file pone.0243333.s004.tif]

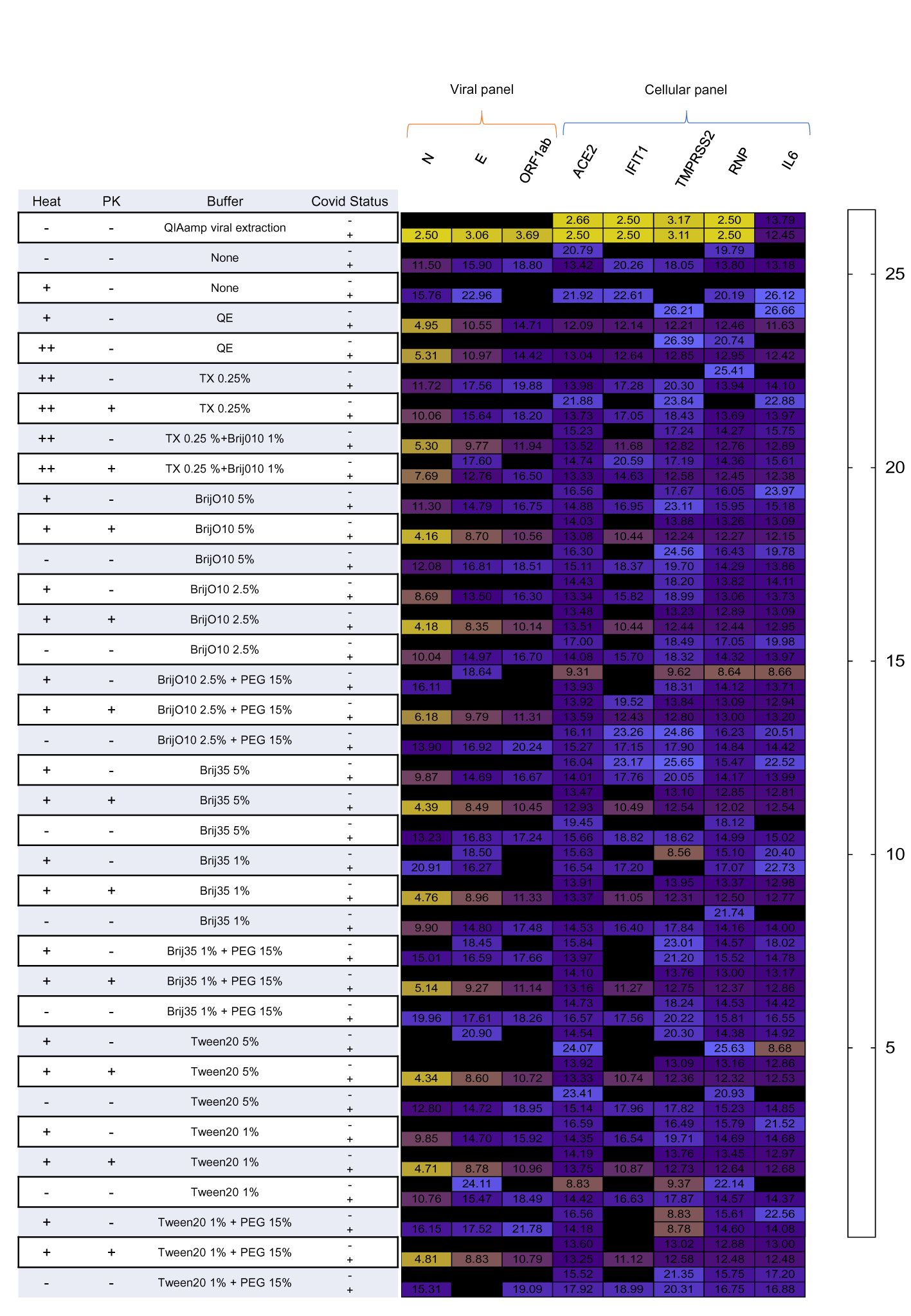

Supplement: S5 Fig — Samples from a positive- or negative-COVID-19 patient collected in a commercial VTM was mixed with different combinations of detergents/emulsifiers, in presence or absence of PK (2 mg/mL) and further heat at 95°C for 5 min or not. Cq values obtained in quadruplicate are presented. Tx: Triton X100; PEG: poly ethylene glycol 600; QE: Quick ExtractTM DNA Extraction Solution. (TIF) [file pone.0243333.s005.tif]
