# Supplementary material for: Versatile and flexible microfluidic qPCR test for high-throughput SARS-CoV-2 and cellular response detection in nasopharyngeal swab samples
Source: PLoS One. 2021 Apr 14;16(4):e0243333. doi: 10.1371/journal.pone.0243333 (PMC8046349; doi:10.1371/journal.pone.0243333)
Supplement: S1 Table — (DOCX) [file pone.0243333.s006.docx]

**S1 Table: List of the micro-RNA tested using the Biomark HD system.**

| Ctrl_miRTC_1 | Hs_miR-223_1 |
| --- | --- |
| Hs_let-7a_2 | Hs_miR-23a_2 |
| Hs_let-7d_1 | Hs_miR-23b_2 |
| Hs_miR-1_2 | Hs_miR-24_1 |
| Hs_miR-103a_1 | Hs_miR-25_1 |
| Hs_miR-10a_2 | Hs_miR-27a_1 |
| Hs_miR-122a_1 | Hs_miR-27b_2 |
| Hs_miR-125a_1 | Hs_miR-28_1 |
| Hs_miR-125b_1 | Hs_miR-296-5p_1 |
| Hs_miR-126_1 | Hs_miR-29a_1 |
| Hs_miR-130a_1 | Hs_miR-29b_1 |
| Hs_miR-133a_2 | Hs_miR-29c_1 |
| Hs_miR-140_1 | Hs_miR-301a_1 |
| Hs_miR-141_1 | Hs_miR-30b_1 |
| Hs_miR-142-3p_2 | Hs_miR-30d_2 |
| Hs_miR-143_1 | Hs_miR-31_1 |
| Hs_miR-146a_1 | Hs_miR-320a_1 |
| Hs_miR-148a_1 | Hs_miR-328-3p_1 |
| Hs_miR-148b_2 | Hs_miR-331_1 |
| Hs_miR-150_1 | Hs_miR-34a_1 |
| Hs_miR-152_1 | Hs_miR-34b_2 |
| Hs_miR-155_2 | Hs_miR-34c_1 |
| Hs_miR-15b_2 | Hs_miR-3620-3p_1 |
| Hs_miR-16_2 | Hs_miR-374a_1 |
| Hs_miR-17_2 | Hs_miR-375_2 |
| Hs_miR-181a_2 | Hs_miR-382_2 |
| Hs_miR-18a*_1 | Hs_miR-409-3p_1 |
| Hs_miR-191_1 | Hs_miR-424_1 |
| Hs_miR-195_1 | Hs_miR-429_1 |
| Hs_miR-197_2 | Hs_miR-4455_1 |
| Hs_miR-199a-3p_1 | Hs_miR-449_1 |
| Hs_miR-200b_3 | Hs_miR-449b_1 |
| Hs_miR-200c_1 | Hs_miR-451_1 |
| Hs_miR-203_1 | Hs_miR-484_1 |
| Hs_miR-204_1 | Hs_miR-486_1 |
| Hs_miR-205_1 | Hs_miR-548b-5p_1 |
| Hs_miR-20b_1 | Hs_miR-574-3p_1 |
| Hs_miR-21_2 | Hs_miR-660_1 |
| Hs_miR-210_1 | Hs_miR-92_1 |
| Hs_miR-214_2 | Hs_miR-93_1 |
| Hs_miR-216a_1 | Hs_miR-98_1 |
| Hs_miR-22_1 | Hs_RNU6-2_11 |
| Hs_miR-221_1 |  |
