# Supplementary material for: Versatile and flexible microfluidic qPCR test for high-throughput SARS-CoV-2 and cellular response detection in nasopharyngeal swab samples
Source: PLoS One. 2021 Apr 14;16(4):e0243333. doi: 10.1371/journal.pone.0243333 (PMC8046349; doi:10.1371/journal.pone.0243333)
Supplement: Attachment — Submitted filename: Response to Reviewers.pdf [file pone.0243333.s007.pdf]

Responses to the reviewers (Responses in bold)

1. Is the manuscript technically sound, and do the data support the conclusions?

The manuscript must describe a technically sound piece of scientific research with data that supports the conclusions. Experiments must have been conducted rigorously, with appropriate controls, replication, and sample sizes. The conclusions must be drawn appropriately based on the data presented.

Reviewer #1: Yes

Reviewer #2: Yes

---

2. Has the statistical analysis been performed appropriately and rigorously?

Reviewer #1: Yes

Reviewer #2: No

No comments mentioned a statistical problem. However, we have reanalyzed the results of the multiple comparisons (Fig.3) using a more appropriate test (ie: one-way ANOVA followed by Kruskal-Wallis test) as mentioned in the Materials and Methods section (lane 261).

---

3. Have the authors made all data underlying the findings in their manuscript fully available?

The PLOS Data policy requires authors to make all data underlying the findings described in their manuscript fully available without restriction, with rare exception (please refer to the Data Availability Statement in the manuscript PDF file). The data should be provided as part of the manuscript or its supporting information, or deposited to a public repository. For example, in addition to summary statistics, the data points behind means, medians and variance measures should be available. If there are restrictions on publicly sharing data—e.g. participant privacy or use of data from a third party—those must be specified.

Reviewer #1: Yes

Reviewer #2: Yes

IPMC, 660 route des Lucioles, Sophia-Antipolis
06560 Valbonne, France
+33 (0)4 93 95 77 77 - Faw +33 (0) 4 93 95 77 08
www.ipmc.cnrs.fr

4. Is the manuscript presented in an intelligible fashion and written in standard English?

PLOS ONE does not copyedit accepted manuscripts, so the language in submitted articles must be clear, correct, and unambiguous. Any typographical or grammatical errors should be corrected at revision, so please note any specific errors here.

Reviewer #1: Yes

Reviewer #2: Yes

---

5. Review Comments to the Author

Reviewer #1: In this study, Dr. Bernard Mari and colleagues addressed a significant question regarding the two major obstacles of current COVID-19 test, the reagent shortage and tedious process of sample preparation. The authors delicately evaluated current approaches with a high-throughput platform in clinical samples. Overall, the data presented in the manuscript are of high quality with several important connections demonstrated, including accuracy and sensitivity. This manuscript should be great interest to a general audience, especially those countries are suffering from the massive COVID-19 test loading. In conclusion, I would suggest the editor directly accept this manuscript without any further revision.

We thank the reviewer for these very positive comments.

Reviewer #2: The authors demonstrated a way to multiplex and bypass the RNA extraction for SARS-CoV-2 detection. The application of the IFC method is new and serve the purpose. However, the sample number of SARS-CoV-2 patients is low and damper the conclusive findings. I have several comments.

Specific comments

1. Increase the sample size of SARS-CoV-2 patients if possible.

We agree with the reviewer that increasing the size of SARS-CoV-19 patient would improve the manuscript. However, this is not possible as our access to patient samples is limited, specifically for direct detection as we receive samples collected and transported in different VTM. As a result, this paper should, in our view, be regarded as a technical report and not necessarily as a fully validated clinical study.

2. Author should explain why to detect miRNA and the importance.

It is unclear, at this stage, whether miRNA detection will be of any importance. However, we felt that being able to demonstrate that high-throughput miRNA detection is possible enriches the manuscript. We feel that this proof of principle adds value to the report and we have decided to keep this section in the revised manuscript.

3. The label in figure 5 second row is quite confusing. Maybe change to VTM. I don't understand the labeling in the figure 5 right part. DP? N? How you calculate sensitivity, by adding?

We have modified the figure 5 (Figure 6 in the revised version of the manuscript) and we feel that this new version is more reader-friendly. The reviewer is right. We have calculated the sensitivity by adding the positive samples with the different probes. However, we omitted to define the acronym "DP" (which stands for double positive). This omission may have confused the reviewer. We have amended the legend of the figure accordingly.

4. I won't say the 11 to 14 out of 17 is high. It is not acceptable for any applications.

We have removed the adjective "high". The new sentence reads: "The R^2 values dropped in a dramatic way for the two direct detergents based assays, with 11 to 14 positive samples detected out of 17" (lanes 469-470).

5. The specificity was not evaluated in the lysis buffer and direct assay. Please revise.

We performed some assays on one negative sample and no-SARS-CoV2-specific signal was obtained in conditions of the Figure 5 (Figure 6 in the revised version). This is now mentioned in the legend of figure 6 in the revised manuscript.

6. The picture quality of the supplementary figures are very poor.

We have provided a new set of supplementary figures of a higher quality.

7. Please check all the labeling in text and figures and be consistent, such as SARS-CoV-2 and COVID-19.

We have performed a few rounds of editing of the paper and are think that the text and figures are consistent.

8. Is there any conflict of interest?

We do not report any conflict of interest (see point number 3 and 4 in the responses to the Editor's comments).
