# Supplementary material for: Versatile and flexible microfluidic qPCR test for high-throughput SARS-CoV-2 and cellular response detection in nasopharyngeal swab samples
Source: PLoS One. 2021 Apr 14;16(4):e0243333. doi: 10.1371/journal.pone.0243333 (PMC8046349; doi:10.1371/journal.pone.0243333)
Supplement: Attachment — Submitted filename: Response to Reviewers2.docx [file pone.0243333.s008.docx]

**Responses to the reviewers (Responses in bold)**

*Reviewer #2: The authors have address most of my comments. There is a few comments still need to be addressed.*

*1. The author should have some sentences or a paragraph to describe the rationales, the findings, and the indications of miRNA. It is not a proper way to explain by merely adding more data.*

**As recommended, we have added a paragraph in the discussion (lanes 523-532) describing the rationale, the findings and the indication of following miRNAs as biomarkers in nasopharyngeal swabs and cited 5 additional references to illustrate this point.**

*2. Please check the SARS-CoV-2 and be consistent. I still see many SARS-CoV2 throughout the manuscript.*

**We have corrected these last typos.**
